# Corrosion and Antifouling Behavior of a New Biocide-Free Antifouling Paint for Ship Hulls Under Both Artificially Simulated and Natural Marine Environment

**DOI:** 10.3390/ma18133095

**Published:** 2025-06-30

**Authors:** Polyxeni Vourna, Pinelopi P. Falara, Evangelos V. Hristoforou, Nikolaos D. Papadopoulos

**Affiliations:** 1 Institute of Nanoscience and Nanotechnology, National Centre for Scientific Research “Demokritos”, 15341 Agia Paraskevi, Greece; 2 School of Chemical Engineering, National Technical University of Athens, 9 Iroon Polytechniou Str., 15780 Zografou, Greece; pin.falara@gmail.com; 3 Institute of Communication and Computer Systems, 15773 Zografou, Greece; hristoforou@ece.ntua.gr; 4 Department of Research and Development, BFP Advanced Technologies G.P., Peristeri Industrial Zone, 12133 Athens, Greece; npapadopoulos@bfp-tech.com

**Keywords:** naval steel, antifouling, anticorrosive, coating, biocide free, environmentally friendly

## Abstract

This study involved covering naval steel samples with a biocide-free, innovative antifouling coating, which were subsequently immersed in either artificial seawater or a Greek maritime environment for durations ranging from 1 to 50 weeks. The objective was to assess the efficacy of the coating as an anticorrosion and antifouling barrier on the steel samples. Non-coated samples were analyzed alongside the coated samples for comparative purposes. The findings indicate that a reduction in coating thickness during static immersion in laboratory settings leads to the removal of precipitated corrosion products, exposing a fresh layer of “pristine” coating. This layer decreases the corrosion rate by almost 90% throughout extended immersion durations. The efficacy of the coating is validated through trials conducted in natural maritime environments, demonstrating an operational performance of 99% for the coated samples after 50 weeks of continuous exposure to seawater. In fact, the coated samples showed only soft fouling, in contrast to the uncoated samples which were characterized by a strong presence of hard fouling within a short period of time after immersion.

## 1. Introduction

Marine biofouling denotes the undesirable colonization of solid artificial structures (e.g., ship hulls, mechanical equipment, or pier pylons) submerged in seawater by marine organisms, including biotic and abiotic dissolved compounds, microorganisms, plants, and animals [1,2,3].

Over 4000 recognized species of biofoulants exist in maritime environments [4]. Biofouling is a multifaceted process including the colonization of an immersed surface by microscopic organisms (microfouling) and bigger species (macrofouling). Microfouling mostly encompasses proteins, bacteria, fungi, viruses, cyanobacteria, protozoa, and microalgae [5]. Macrofoulants encompass macroalgae (multicellular creatures) and macroinvertebrates (calcareous hard fouling organisms: acorn barnacles, tubeworms, mussels bryozoans, sedentary polychaetes, bivalves, ascidians and soft-fouling organisms: hydroids, solidary anemones, sponges, tunicates) [6].

Two models have been presented to elucidate the process of biofouling evolution on submerged surfaces in marine environments: a classical model [6] and a dynamic model [6]. The traditional colonization model delineates the intricate life cycle of biofouling as a sequence of four primary stages in the colonization of marine organisms [7,8,9,10]. In the dynamic model of biofouling, the lack of one colonization stage does not inhibit the emergence of another stage, as colonization is significantly influenced by the variety and quantity of organisms [5,6]. Nonetheless, the requirement for the establishment of an initial colonization layer persists in the two proposed models for elucidating the biofouling process of surfaces submerged in seawater [5].

The biological deposition of fouling on ship hulls is a global issue [11], as it is associated with interconnected consequences:

- Heightened hull corrosion rates result in augmented surface roughness [12], causing drag resistance to increase [13] by as much as 70% during the initial colonization phases [14] and up to 86% in subsequent stages [15];
- Elevated friction resistance may lead to a decrease in cruising speed [16];
- To sustain the preset speed, greater fuel consumption is necessitated [17], potentially reaching 40% [18];
- Excessive fuel consumption results in economic and environmental repercussions: Escalation in transportation expenses [19] and greenhouse gas emissions that could threaten global warming [20];
- The accumulation of marine organisms on the ship’s hull necessitates more frequent dry-docking [21], leading to heightened maintenance expenses [22] and diminished voyage duration [23];
- The recurrent dry-docking procedure generates substantial toxic waste, detrimental to the environment [24];
- Vessels transport invasive organisms globally, potentially resulting in the bio-invasion of non-native species in marine ecosystems devoid of natural enemies [25].
- Τhe proliferation of contaminating organisms modifies the interface between the metal surface and its surroundings, resulting in the onset of microbially induced corrosion (MIC) of the ship’s hull [21,26].

**Figure 1:**
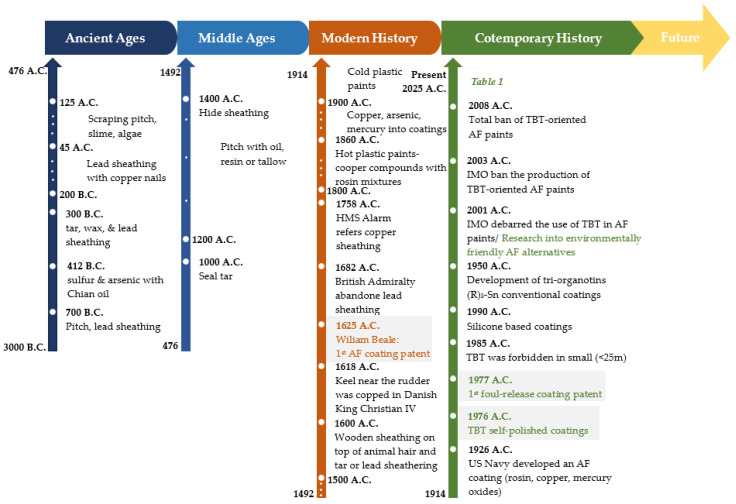
Historical timeline of antifouling coatings development on ship hulls, highlighting key milestones. The color coding signifies the different historical periods.

The attempt of preventing biofouling through the development of antifouling treatments dates back to prehistoric times and the early stages of maritime transportation of people and goods [27]. Figure 1 presents a timeline that summarizes the evolution of antifouling technologies from ancient times to the contemporary day, as evidenced by an analysis of review articles pertaining to antifouling technology during the past three decades [1,3,6,18,20,21,27,28,29,30,31,32,33].

Table 1 delineates the advantages and disadvantages of the different technologies, underscoring the urgency of addressing biofouling and the necessity for extensive research by the scientific community into the development of novel, efficient, eco-friendly antifouling coatings for ship hulls [20,28,34,35,36].

By evaluating each contemporary antifouling technology over the past 25 years against the criteria outlined in Table 2, a comparison table is generated.

**Table 2:** Comparison table of modern antifouling technologies.

Criterion	Self-Polishing Copolymers	Silicon/ Fluoropolymer Coatings	Biomimetic Surfaces	Robotic Hull Cleaning	UV/ Electromechanical Systems
Antifouling Efficacy	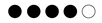	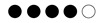	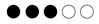	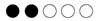 (removes, not prevents)	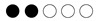 (still in development)
Environmental Compliance	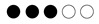 (low tox biocides)	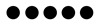 (no biocides)	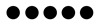 (no chemicals)	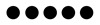	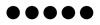
Durability/Mechanical Resistance	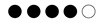	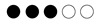 (softer coatings)	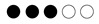	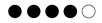	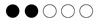 (vulnerable tech)
Ease of Application/Maintenance	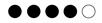	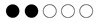 (requires special prep.)	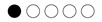 (experimental, costly)	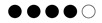 (operational only)	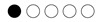 (lab-scale)
Cost Effectiveness	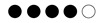	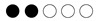 (high initial cost)	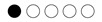 (R&D phase)	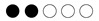 (high upkeep)	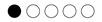 (experimental)
Fuel Efficiency/ Drag Reduction	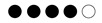	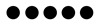 (very smooth)	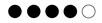	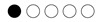 (does not alert surface)	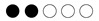
Smart/ Innovative Features	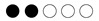	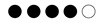 (self-cleaning)	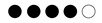 (bio-inspired)	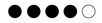 (autonomous)	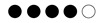 (novel tech)
Best for	Cargo, tankers, general fleet	High-performance, eco-focused ships	Research, green shipping demos	All ship types during port	Future ships (concept/prototype)

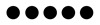
 = excellent; 
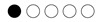
 = poor.

Table 1 and Table 2 clearly indicate that the advancement of antifouling technology for ship hulls, which would significantly mitigate fouling, remains a contentious subject today. Bio-disposal results in significant economic and energy losses, along with numerous severe ecological issues, and should be avoided. Conversely, traditional antifouling methods, such as copper based, frequently exhibit toxicity and can inflict significant environmental harm. Moreover, contemporary, eco-friendly alternatives remain less effective and are not widely adopted.

This study assessed a non-biocidal, fouling-resistant, amphiphilic nanostructured coating via static immersion in both laboratory and in situ conditions, in light of the urgent need to combat biofouling and the requirement for comprehensive research by the scientific community to create new, effective, and environmentally sustainable antifouling coatings for ship hulls.

To achieve this objective, naval steel samples were coated with the antifouling coating and underwent laboratory immersion tests in artificial seawater (ASW), as well as static exposure at three distinct locations in Greek waters (Rafina, Kalamata, Andros), over a 50 weeks field study. At multiple time intervals (1 week, 2 weeks, 8 weeks, 17 weeks, 26 weeks and 50 weeks), measurements of mass alterations in submerged samples in artificial seawater were conducted, alongside evaluations of coating efficacy via visual inspection of the in situ submerged panels. The aim of this study was to assess the anticorrosion and antifouling efficacy of the AF coating.

## 2. Materials and Methods

### 2.1. Antifouling Coating

#### 2.1.1. The Synthesis Procedure

The antifouling coating is produced using a novel approach that synthesizes a water-soluble resin matrix with heterogeneously dispersed polyaniline (PAni) nanorods and chemically modified multiwall carbon nanotubes (ΜMWCNTs) [37]. The resultant multifunctional hydrophobic coating demonstrates electrical anisotropy and serves as either an antifouling protective coating during dry-docking or as a non-stick coating with fouling release properties during operational settings.

The synthesis protocol comprised the subsequent steps: (1) synthesis of PAni nanorods via chemical oxidation of aniline utilizing ammonium peroxydisulfate (APS, (NH_4_)_2_S_2_O_8_) as the oxidizing agent, (2) functionalization of conductive doped polyaniline nanorods with photocatalytic titanium dioxide (TiO_2_) nanoparticles, (3) coating the inner surface of multiwalled carbon nanotubes (MWCNTs) with magnetite (Fe_3_O_4_) nanoparticles, (4) dispersion of PAni/TiO_2_ and MWCNTs/Fe_3_O_4_ in a water-soluble resin at a mass ratio of 1:1:3, and (5) incorporation of additives.

Initially, 10 mmol of an acidic solution (1 M HCl) was dissolved with 80 mL of distilled water, followed by the quick addition of 10 mmol of aniline monomer (Ani) to the oxidant solution while stirring vigorously for 30 min. 40 mL of distilled water was gradually incorporated into the HCl/Ani mixture while stirring for approximately 5 min, and subsequently allowed to stand for 12 h. Subsequently, 0.20 g of TiO_2_ was meticulously disseminated in the Ani/HCl mixture using ultrasonication for a duration of 24 h. The polymerization of aniline was conducted by gradually introducing 3.82 g of APS into the suspension. The nanocomposite was centrifuged to extract the product and subsequently washed three times with distilled water and methanol, respectively. The product underwent drying for 24 h at 80 °C, resulting in a yield of 5.21 g of PANI coated with TiO_2_.

In a 1000 mL round-bottom flask, 1 g of MWCNTs was dispersed in 7 M nitric acid (HNO_3_) and subjected to reflux at 85 °C for 5 h. Subsequently, three centrifugation cycles, each lasting 10 min, were conducted until the pH attained 5. The MWCNTs were subjected to vacuum filtering to attain a final pH equivalent to that of deionized water, followed by drying the samples at 75 °C for 2.5 h. 200 mg of dry MWCNTs were disseminated in deionized water using ultrasound for 30 min. Solutions of FeCl_3_·6H_2_O and FeCl_2_·4H_2_O in 0.5 M HCl were gradually introduced to this solution in a molar ratio of Fe^3+^:Fe^2+^ equals to 2:1. The proportion of iron precursor salt to MWCNTs was 4:1. A 2 M ammonia solution was incrementally added to the suspension until the pH attained 10. The mixture was stirred mechanically for 1 h at 50 °C to confirm the completion of the reaction. The solution was subsequently rinsed with absolute ethanol and deionized water to attain a neutral pH, and then dried for 48 h at 40 °C.

The antifouling coating comprises a water-soluble resin matrix incorporating PAni/TiO_2_ and MWCNTs/Fe_3_O_4_ nanocomposites in a mass ratio of 3:1:1, along with supplementary additives. 

#### 2.1.2. Characterization of Nanocomposites

In order to study microstructure, chemical composition and physical properties of the antifouling coatings various analytical techniques were employed. More specifically, to investigate the morphology elemental analysis of synthesized PAni/TiO_2_ and MWCNTs/Fe_3_O_4_ nanocomposites, a JEOL JSM-6490LV scanning electron microscope (JEOL, Tokyo, Japan) (SEM) with an EDS analyzer was used. The preparation of PANI-TiO_2_ and MWCNFs-Fe_3_O_4_ samples took place by sprinkling ground powder onto carbon tape attached to aluminum mounts. Transmission Electron Microscope (TEM) bright field images were performed using JEOL 2100 HR Microscope (JEOL, Tokyo, Japan) with an accelerating voltage of 80 kV. To prepare samples for TEM analysis, small amount of each nanocomposite was dispersed in deionized water using ultrasonicator for 5 min, and then a drop of dispersion was deposited on a carbon coated grid (Cu Mesh 300) and placed in specimen chamber.

Spectroscopic analysis of samples was performed using Fourier Transform Infrared Spectroscopy (FTIR) on Bruker 27 IR equipment (Bruker, Billerica, MA, USA) in the 500–3500 cm^−1^ range 10 kHz scan speed.

The conductivity of PANI/TiO_2_ composites was measured by the four-point probe method (Keithley 2000 multimeter, Keithley 220 programmable current source, and Signatone probes).

### 2.2. Panel Preparation

Naval steel (EH36) panels were lightly sanded and then were cleaned with deionized water and ethanol. After drying at room temperature, were manually coated with two layers of the antifouling paint without an initial primer or top coat application.

**Figure 2:**
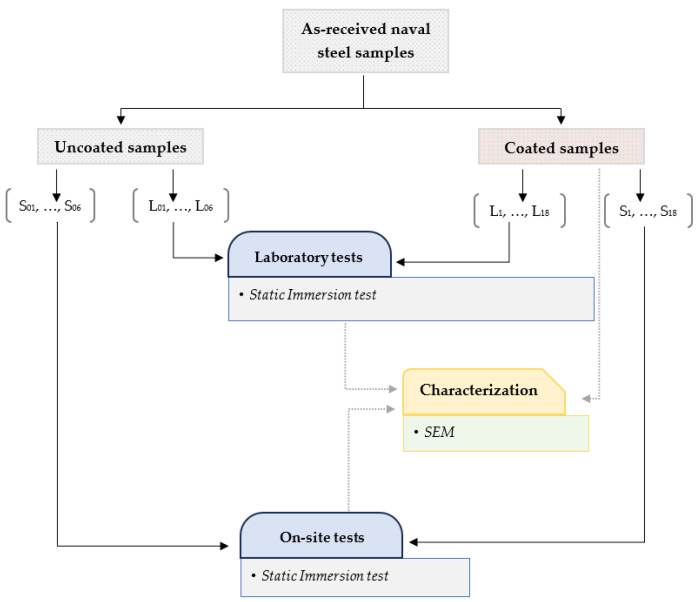
Diagram illustrating the experimental protocol for the corrosion evaluation of uncoated and coated steels.

Two sets of coated naval steel panels were prepared: one set for corrosion assessments in controlled laboratory conditions (4 cm^L^ × 4 cm^W^ × 2 mm^T^) and another set for evaluating corrosion resistance in situ under actual seawater conditions (20 cm^L^ × 20 cm^W^ × 2 mm^T^) (Figure 2).

### 2.3. Laboratory Static Immersion Tests

Figure 2 illustrates that the long-term performance of the antifouling paint was initially assessed in laboratory conditions by static immersion testing in artificial seawater (ASW) [38]. The static laboratory immersion tests in ASW complied with the ASTM D1141 [39] and ASTM G 31-72 [40] standards.

**Figure 3:**
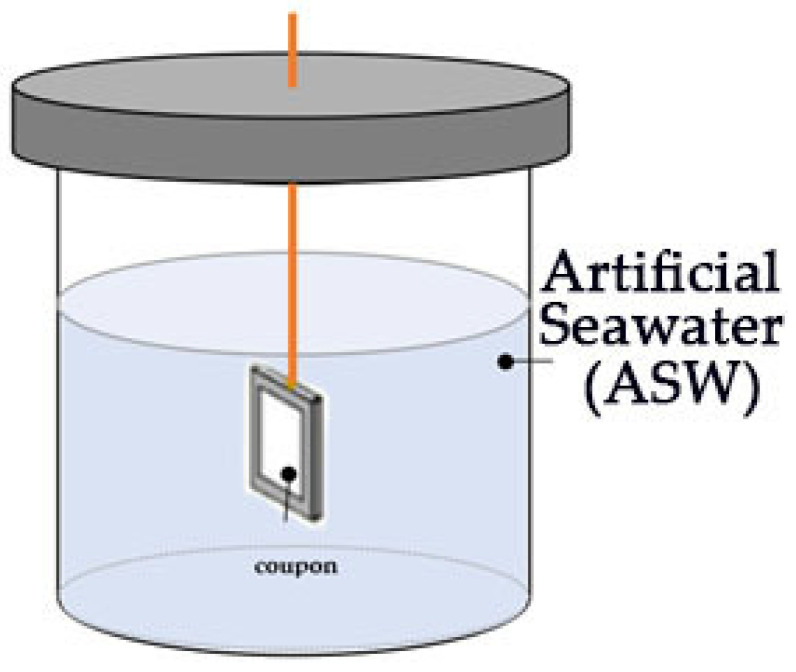
Apparatus used in the laboratory static immersion test. The orange line denotes a non-conductive coupon retention cord.

Six naval steel samples were covered with antifouling paint for the laboratory tests (L0–L6 in Figure 2). Each coupon was positioned within an appropriately designed container (Figure 3) that was filled with ASW. The coupons were positioned at a depth of 3.5 cm below the surface of the ASW.

The immersion duration differed among the six samples and is presented in Table 3. Static immersion studies were also conducted on uncoated materials (L00–L06 in Figure 2) for the same duration for comparative analysis. The temperature was consistently maintained at 27 °C during the laboratory immersion tests.

Upon concluding their duration in ASW, the samples were meticulously removed from their container and air-dried. Each sample was subsequently weighed using digital precision scales to ascertain the weight variation before and after immersion, serving as an indicator for assessing the corrosion rate of naval steel [40]. The mass of each sample was measured thrice, and the mean was documented. The methodology for assessing the corrosion rate by the weight variation in the examined sample is detailed elsewhere [38]. A morphological analysis of the surface and cross-section of the samples post-immersion was conducted utilizing a scanning electron microscope (SEM). The percentage covering of the surface with corrosion products was assessed using (Image-Pro Analyzer software (Image-Pro® Plus Version 7.0 for Windows™), aided by the acquired photos. The aforementioned measurements were conducted on both coated and uncoated steel samples.

To compute the percentage of an area in a picture utilizing Image-Pro Analyzer, initially delineate the area of interest by creating a freehand selection around the corroded region. Subsequently, with the software’s integrated features [41], it may quantify the area and compute its percentage relative to the overall area of the image.

The conductivity of the antifouling coating was verified by Atomic Force Microscope equipped with an extended Tunneling Atomic Force Microscopy (TUNA) module. For conductivity mapping, the current sensitivity was set to 1 nA/V.

The water contact angles of the coated sample subjected to static laboratory immersion tests in ASW were assessed utilizing a contact angle goniometer (DSA 10-MK2, A. Krüss Optronic GmBH, Humburg, Germany). The coated samples were dried in ambient conditions before the measurements were conducted. DI water was dropped on three different places of the coated surface and the contact angles were measured at 30 s intervals for a total of 180 s. The mean values from three measurements were computed and documented.

### 2.4. In Situ Static Immersion Tests

**Figure 4:**
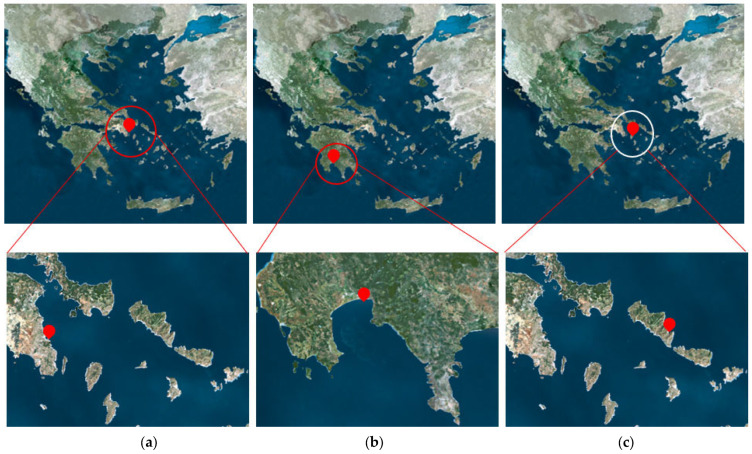
Location and satellite photos of the (**a**) Rafina, (**b**) Kalamata, and (**c**) Andro exposure sites for coated and uncoated naval steel panels. Red pins on a map indicate the precise location of the specific places.

To perform static immersion tests in seawater under real conditions, 18 naval steel samples were gathered and categorized into three groups of six samples each. In early May 2024, each sample group was immersed at a distinct location along the Greek shoreline (Figure 4). All samples in the collection were collected at six specified time intervals: 1 week, 2 weeks, 8 weeks, 17 weeks, 26 weeks, and 50 weeks. Subsequent to extraction, the samples were collected from the marine environment and maintained in the laboratory at room temperature for five days. Alongside the coated samples, uncoated steel samples were concurrently immersed at same time intervals to facilitate a comparison of biofouling rates. The samples were designated based on the methodology of the static immersion laboratory tests (Table 3), with the exception that the letter L was substituted with the letter S.

Each sample extracted from the immersion site after the specified duration in seawater was rinsed with deionized water and ethanol, and thereafter dried at ambient temperature. The macroscopic examination of the samples revealed the nature of the deposited micro- or macrofoulants. Likewise, the analysis of the acquired pictures allowed for the estimation of the biofilm volume fraction in both coated and uncoated steel samples.

The assessment of biofouling in the collected coated samples was performed by measuring the Foul Resistance (FR) and the Physical Data Rating (PDR) in accordance with the standards set out in ASTM D 3623 [42] and ASTM D 6990 [43].

The spectrum of FR values extends from 0 to 100. A score of 100 indicates a coated surface devoid of adhering biological foulants. In macro-colonization, the overall percentage of surface covered is deducted from 100, resulting in the FR representing the percentage of the surface devoid of fouling. As per ASTM D 6990, a rating of 99 indicates a paint layer devoid of macrofouling but either partially or wholly obscured by microfouling, irrespective of the surface coverage %.

The spectrum of PDR values spans from 0 to 100. A coated specimen exhibiting no physical deterioration is designated as 100. Upon cleaning the surface, the cumulative wear rates will be deducted from 100, yielding a figure that indicates the natural wear of the residual permeable coating. The Overall Performance (OP) score was the lesser value between the FR and PDR metrics.

## 3. Results

### 3.1. Characterization of the Antifouling Nanocomposites

**Figure 5:**
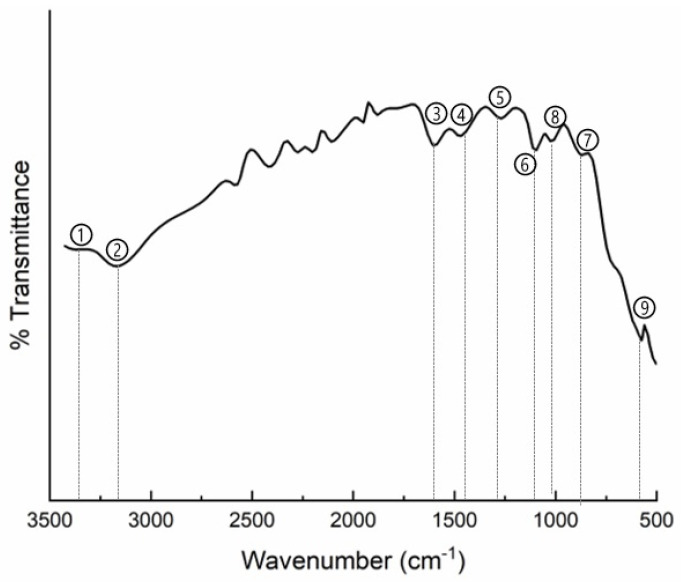
FTIR spectra of PAni/TiO_2_ nanocomposite.

FTIR spectroscopy studies were conducted to ascertain the conducting state of PAni and to examine the interactions between the polymer and titania in the PAni/TiO_2_ nanocomposite (Figure 5). The FTIR spectra of the PAni/TiO_2_ nanocomposite were recorded within the region of 500–3500 cm^−1^.

The FTIR spectrum of the PAni/TiO_2_ nanocomposite revealed all the main peaks of PAni along with the corresponding peaks of TiO_2_ (Table 4).

The presence of the principal peaks of PAni (C=C and C–N peaks), along with the Ti–O–Ti peak, signifies the incorporation of TiO_2_ into PAni. The effective interaction between TiO_2_ and PAni resulted in the coating of the nanotubes with TiO_2_ facilitated by the coordination of the nitrogen in PAni with TiO_2_ [44].

**Figure 6:**
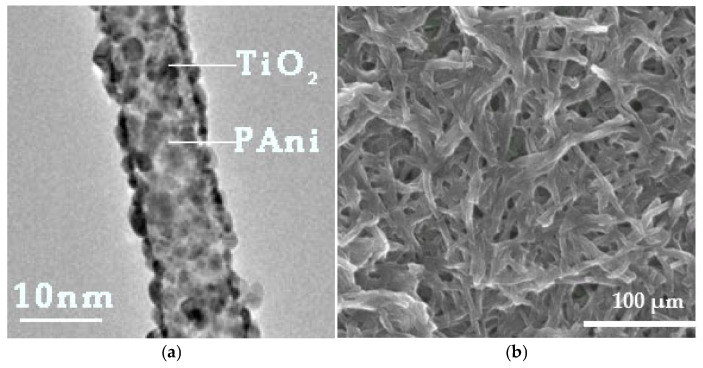
(**a**) TEM’s bright field image and (**b**) SEM micrograph of functionalized PAni nanorods with TiO_2_ NPs.

A small quantity of nanotubes was observed by TEM. The bright field image further confirmed the successful decoration of titanium oxide nanoparticles at the exterior of PAni’s nanotubes (Figure 6a). SEM micrographs illustrate nanocomposite PAni/TiO_2_ aggregates with an average size ranging from 200 to 700 μm (Figure 6b). Notably, pure PAni or TiO_2_ aggregates were undetected in the SEM analysis, suggesting that TiO_2_ in the PAni/TiO_2_ composite mostly decorates the surface of PAni.

The synergistic impact of PAni/TiO_2_ not only verifies the presence of titania to the polymer but also indicates that PAni is in its conductive state, as interactions between TiO_2_ necessitate the polymer’s conductivity [50]. Conductivity measurements revealed that the conductivity of pure PAni exhibited a marginal decrease within the PAni-TiO_2_ nanostructure, from 6.72 × 10^−5^ S cm^−1^ to 6.15 × 10^−5^ S cm^−1^, respectively. This alteration is ascribed to the higher concentration of defects [51].

**Figure 7:**
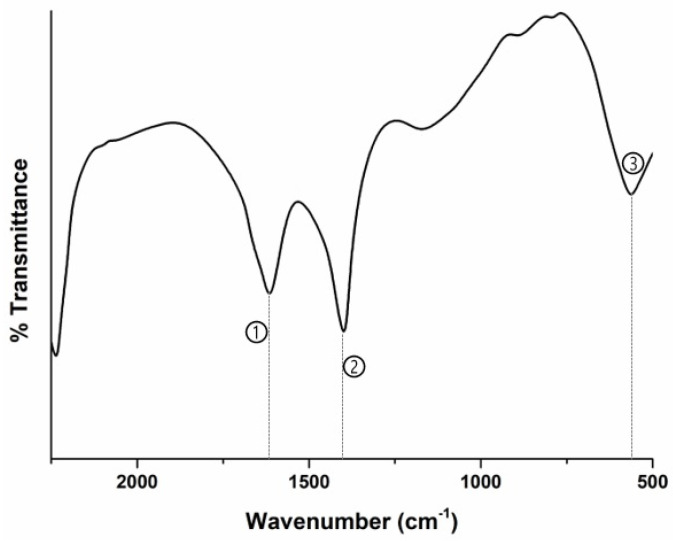
FTIR spectra of the modified MWCNFs with Fe_3_O_4_.

The modification of MWCNFs with Fe_3_O_4_ was confirmed by using FTIR spectroscopy (Figure 7). The spectra revealed that there are several peaks presenting the functional groups of the MWCNT and the Fe_3_O_4_ (Table 5). Based on this characterization, it can be stated that carboxyl groups were successfully generated on the surface of the MWCNTs and attached the Fe_3_O_4_ nanoparticles.

**Figure 8:**
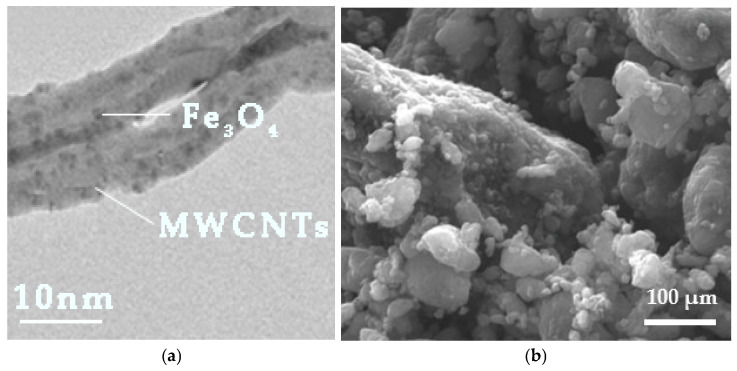
(**a**) TEM’s bright field image and (**b**) SEM micrograph of functionalized carbon nanotubes with magnetite NPs.

Following the magnetic treatment of the MWCNTs, TEM bright field images distinctly revealed that the MWCNTs were adorned with Fe_3_O_4_ nanoparticles. A quantity of isolated nanoparticles appeared to have been randomly adsorbed onto the external surfaces of the MWCNTs (Figure 8a). SEM micrographs (Figure 8b) revealed that clustered MWCNTs/Fe_3_O_4_ complexes were predominantly spherical, enveloped by a dense magnetite nanoparticle structure exhibiting an uneven morphology.

**Figure 9:**
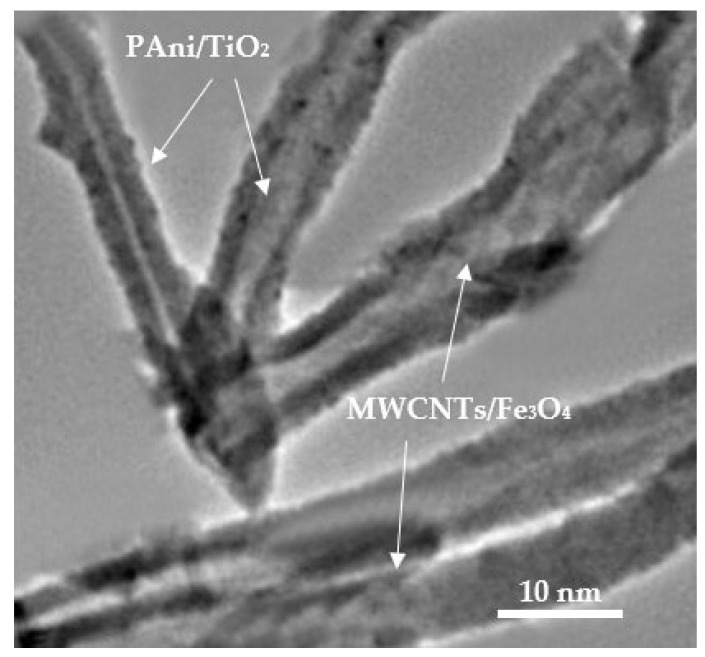
TEM’s bright field image antifouling coating.

The resulting antifouling coating exhibited modified polyaniline nanorods predominantly arranged vertically, whilst the functionalized carbon nanotubes with magnetite nanoparticles were developed in horizontally oriented overlaid layers (Figure 9).

**Figure 10:**
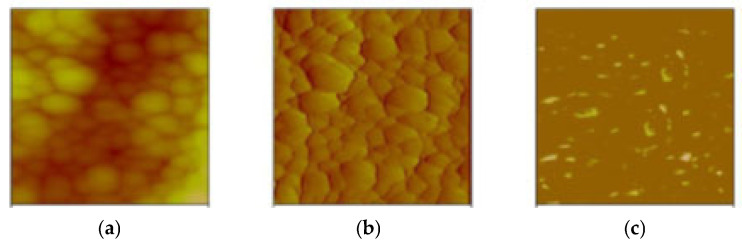
(**a**) Conductive AFM mapping of the antifouling coating, (**b**) topography image, and (**c**) current map at 10 mV DC sample bias.

The integration of conductive elements (PAni, MWCNTs) and their aligned arrangement in the final antifouling coating (Figure 9) enabled the development of structures demonstrating directional conductivity. The modulation of the conductivity properties of the final antifouling coating was performed using conductivity AFM (C-AFM) measurements (Figure 10). The existing flow is depicted by the black patches and/or dark areas in Figure 10a. The current flow is primarily focused inside the assessed surface of an enlarged region. The conductivity patches and topographic relief have been defined by extracting the current flow picture (Figure 10b) and the topographic image (Figure 10c) from the original acquired image (Figure 10a). The average roughness of the coating was determined to be approximately 200 nm (Figure 10b), while the simultaneously acquired current picture confirmed a surface current flow on the paint (Figure 10b). The current spots primarily manifest at the peripheries of the cavities of the surface protrusions, enabling effective electron transport along the antifouling coating (Figure 10b).

### 3.2. Corrosion Tests of Antifouling Coating

#### Static Immersion Tests

**Figure 11:**
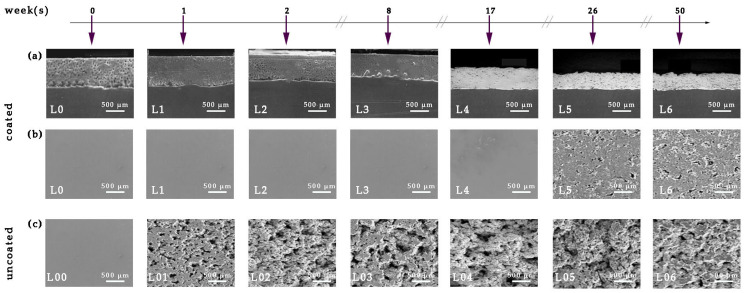
SEM micrographs of (**a**,**b**) coated and (**c**) uncoated naval steel samples following their static immersion in ASW. (**a**) Cross-section and (**b**,**c**) surface images. The designation in the lower left corner of each subfigures (LX or L0X, where X ranges from 1 to 6) corresponds to the sample names shown in Table 3.

Figure 11 presents the SEM images of the surface and cross-section of the coated samples prior (0 weeks) to and following immersion in ASW, for various immersion times. Surface SEM’s micrographs of the uncoated steel samples are presented for comparative analysis.

Analysis of the cross-section micrographs of the coated specimens (Figure 11a(L0)–(L6)) reveals that, despite the absence of a primer or binder layer, the coating remains continuous and conforms to the topographical contours of the steel surface. Complete uniformity with the substrate signifies exceptional adhesive characteristics to the steel substrate. The antifouling coating layer is notably devoid of flaws, voids, or micro-cracks during its formation and is uniformly distributed across the surface of the metal substrate in the observed locations (Figure 11a). The coating layer is critically devoid of trapped pores, as well as longitudinal or transverse micro-cracks that could facilitate ASW adsorption and compromise the barrier properties of the coating. The antifouling coating–steel interface remains intact after 50 weeks of exposure to ASW (Figure 11a(L6)).

**Figure 12:**
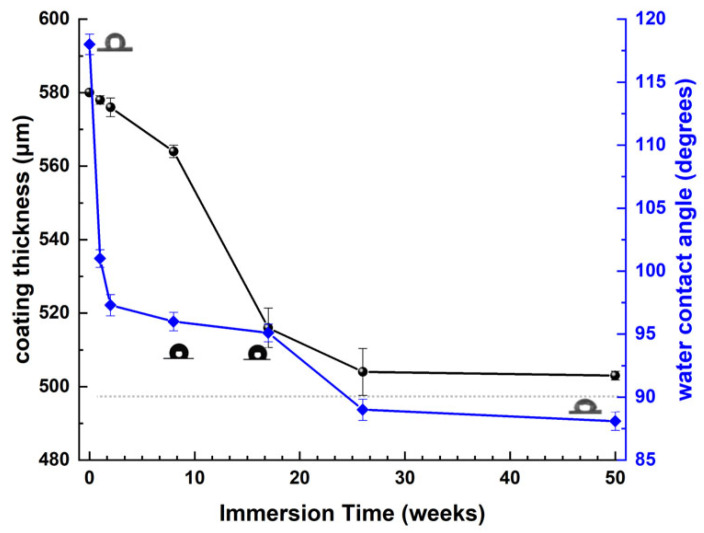
Measurements of coating thickness and water contact angle (WCA) as a function of immersion duration in artificial seawater (ASW). The dotted line at 90 degrees signifies the change from hydrophobic behavior (WCA > 90 degrees) to hydrophilic behavior (WCA < 90 degrees). Representative digital photos of water droplets on the coated steel samples are also presented in the figure.

The coating thickness initially exhibits a minor reduction as the immersion duration extends to 8 weeks (Figure 12). The reduction in thickness with immersion time becomes increasingly evident until the 26th week of static immersion, which can be ascribed to the dissolving of the resin in artificial seawater owing to its water-soluble characteristics. During extended immersion times (26–50 weeks), the coating thickness remained virtually constant at approximately 510 μm.

Figure 12 illustrates the variation in the water contact angle (WCA) of the coated sample surface as the immersion period in artificial seawater (ASW) rises. During the preliminary phases of the coated sample’s immersion in ASW, the elevated contact angle is attributed to the hydrophobic characteristics of PAni [55] and MWCNTs [56,57]. As the immersion time in ASW extends and the resin dissolves in water due to its water-soluble properties, the hydrophilic structures of TiO_2_ και Fe_3_O_4_ are exposed, resulting in a reduced contact angle.

The coated steel specimens with antifouling coating exhibited initial indications of corrosion products after 17 weeks of continuous exposure to ASW (Figure 11b(L4)), whereas cracks and holes were not observed even after 50 weeks of exposure to ASW (Figure 11b(L6)). Conversely, examination of the surfaces of the uncoated samples (Figure 11c(L00)–(L06)) following the prolonged laboratory immersion test in ASW reveals that surface corrosion occurs within a brief immersion period (Figure 11c(L01)). As the immersion period in ASW extends, the initial corrosion product patches enlarge and become prevalent across the majority of the metal surface, with the emergence of holes and cracks (Figure 11c(L02)). After 8 weeks of immersion, the corrosion product aggregates had entirely enveloped the steel surface (Figure 11c(L03)). During extended static immersion periods in ASW (Figure 11c(L04)–(L06)), the corroded surface exhibits a significant accumulation of irregular network formations of corrosion products and a prominent topographical relief.

**Figure 13:**
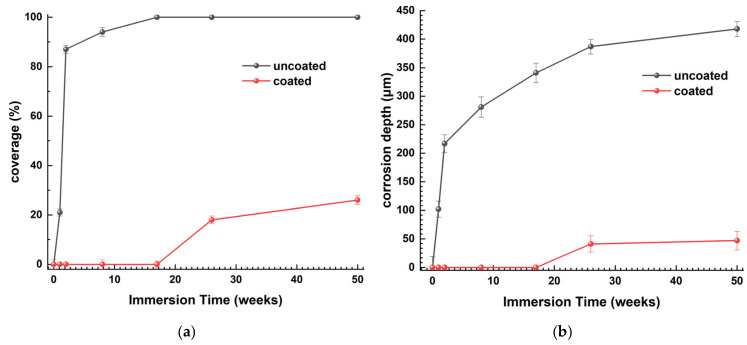
(**a**) Percentage of surface coverage with foulants on samples immersed in artificial seawater (ASW) and (**b**) corrosion depth of corrosion products as a function of immersion duration in ASW for both coated and uncoated naval steel samples.

Figure 13 distinctly demonstrates the protective efficacy of the antifouling coating. With an increase in immersion time in ASW, the proportion of corrosion products on the surface of naval steel stays comparatively low relative to the uncoated samples (Figure 13a). Figure 13b illustrates the recorded corrosion depths for both coated and untreated steel specimens. The “unprotected” steel specimen exhibited substantial corrosion, impairing the functionality of the metal substrate.

In order to clarify the protective anticorrosion efficacy of the antifouling coating, the corrosion rates of both coated and untreated naval steel specimens were assessed by quantifying weight loss, a method noted for its simplicity, accuracy, and dependable reproducibility of results [38,58]. Table 6 illustrates the variation in weight and corrosion rate of the steel specimens over a 50-week immersion period in artificial seawater.

The lack of porosity, along with the adhesion retention to the steel substrate (Figure 11), improves the corrosion resistance of the epoxy coating and validates the barrier characteristics of the antifouling paint (Table 6). After one week of exposure to ASW, the corrosion rate of 21.401 mm/y in the uncoated specimen is reduced to zero in the coated specimen, indicating a 100% decrease. The substantial decrease in the corrosion rate is sustained after 2 weeks of exposure, achieving a value of 99.986% (from 14.996 mm/y to 0.002 mm/y). The corrosion rate reduction percentages for typical immersion durations of 8 and 17 weeks are 99.989% (decreasing from 9.597 mm/y to 0.001 mm/y) and 99.961% (decreasing from 6.273 mm/y to 0.002 mm/y), respectively. Finally, at high retention times this percentage ranges from 95.362% for 26 weeks retention (from 4.373 mm/y to 0.203 mm/y) and 91.835% for 50 weeks retention (from 2.328 mm/y to 0.190 mm/y).

Table 6 demonstrates that the decrease in metal mass (w) and the percentage weight change (w%) increased progressively with extended immersion time in the uncoated steel specimens. The corrosion rate (CR) decreases exponentially with immersion duration, exhibiting significant rates in the initial weeks and subsequently stabilizing after 26 weeks, suggesting that the corrosion layer acts as a protective barrier for naval steel. The immersion of uncoated steel samples in ASW instigates detrimental and unintended corrosion of the surface at a rate of 21.4 mm/y. As immersion duration increases, the surface coverage rises (Figure 13a) and the corrosion thickness intensifies (Figure 12), leading to a decrease in the corrosion rate from 15 mm/y to 6.3 mm/y. Upon complete corrosion of the steel surface (100% coverage in Figure 13a) and the stabilization of corrosion product thickness (Figure 13b), the corrosion rate shows a slight decrease of 2 mm/y; however, it continues to cause surface damage due to the presence of micro- and macro-pits (Figure 11c(L05)–(L06)).

In contrast, the weight change (w) for the coated specimens is negligible up to 17 weeks of immersion, and after 50 weeks in artificial seawater, the weight has decreased by merely 1.7%. Until the 17th week of immersion, absence of notable weight change yields a corrosion rate of zero; thereafter, for a duration of up to 50 weeks in artificial seawater, the corrosion rate remains remarkably low and almost constant. The coated samples with the antifouling coating demonstrate outstanding corrosion resistance on naval steel surfaces. The negligible weight alteration and the ease of removing corrosion products from the coating’s surface demonstrate its superior anti-stick properties and corrosion resistance.

### 3.3. On-Site Immersion of Steels in Real Seawater

The nature, characteristics and intensity of fouling can differ depending on the duration of a metal surface’s exposure to seawater and the site of immersion. Consequently, the coated surfaces were evaluated for performance under different fouling conditions in three distinct locations: Rafina, Kalamata, and Andro.

**Figure 14:**
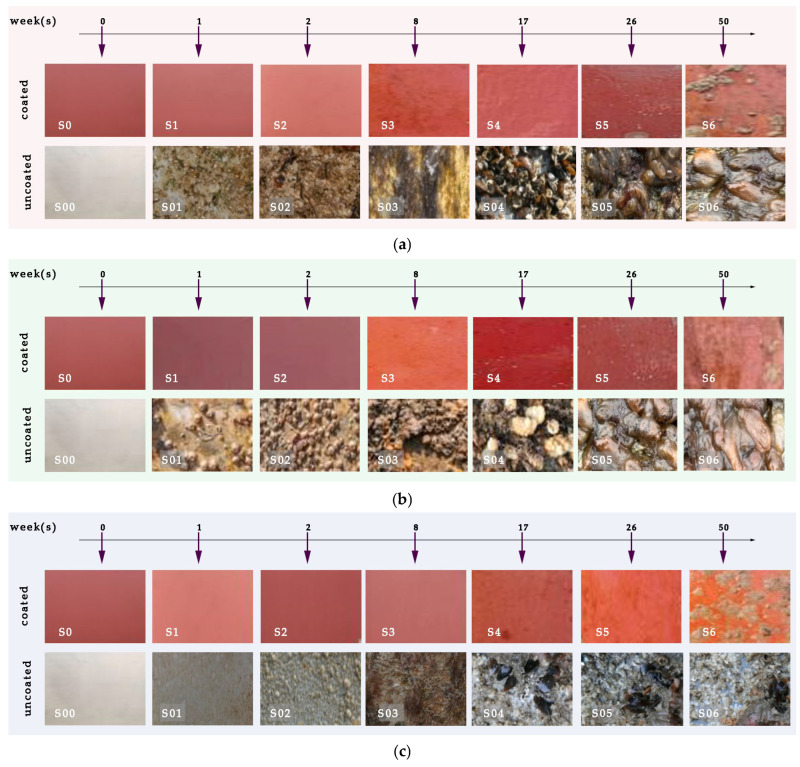
Indictive digital photographs of coated and uncoated naval steel panels submerged at seawater at three locations: (**a**) Rafina, (**b**) Kalamata, and (**c**) Andros.

Figure 14 displays macroscopic digital images of both coated and uncoated naval steel samples, intended for visual assessment at the time intervals outlined in Table 3. The macroscopic analysis of the photos reveals notable and substantial differences in biofouling across the three locations (Rafina, Kalamata, and Andro) concerning foulant frequency, colonizing species, and the timing of their emergence.

All coated naval steel samples demonstrated the presence of both incipient and advanced slime starting from the 17th week of immersion, with the exception of the Andros Sea, where slime became apparent only after the 26th week of immersion in seawater. This is likely attributable to the modest marine fouling that defines the region. At prolonged immersion durations (exceeding 26 weeks), the antifouling coating failed to inhibit soft microfouling. Nonetheless, the quantity of preserved microfouling organisms was little. No macrofouling of the coated sample surface was found at any of the three selected immersion sites (Rafina, Kalamata, and Andro).

The predominant species responsible for surface fouling on the uncoated naval steel samples were tunicates, bryozoans, branching algae, and spiny algae, with hard-shelled organisms appearing in all cases following the 8th week of immersion. Following 17 weeks of exposure to seawater, the pictures in Figure 14 indicate minimal visual distinctions across the selected immersion sites.

**Figure 15:**
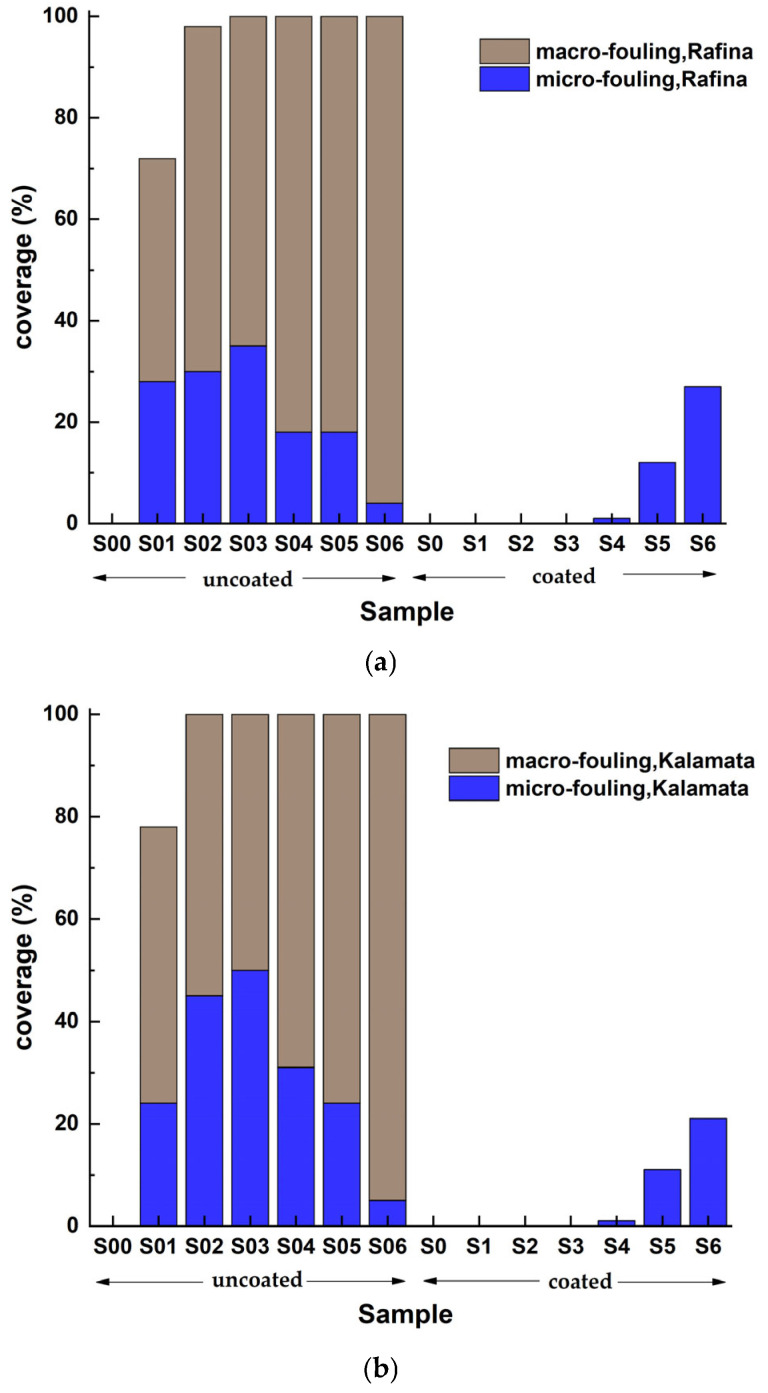

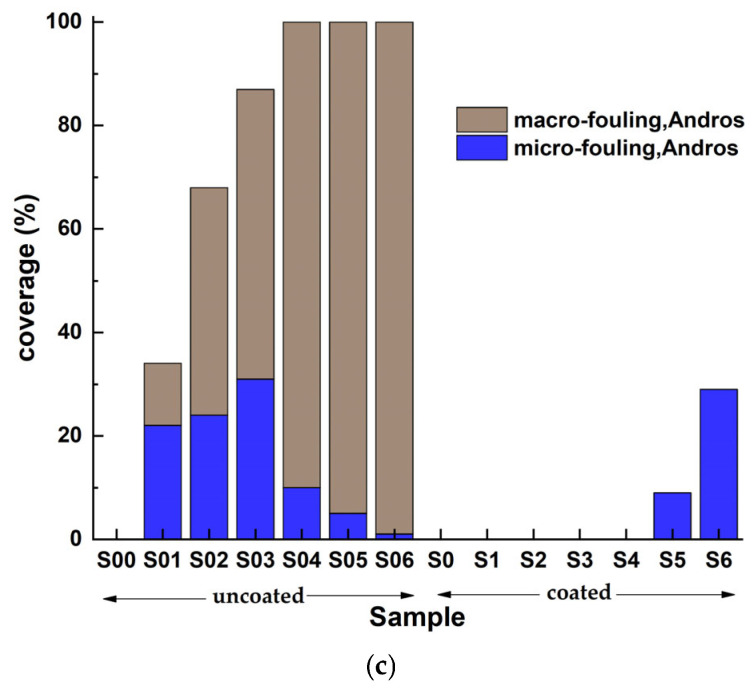
Micro- and macrofouling on uncoated and coated naval steel samples submerged at three locations: (**a**) Rafina, (**b**) Kalamata, and (**c**) Andros.

The visual examination of coated and uncoated samples, considering edge effects, led to the assessment of the percentage coverage of the exposed surface obscured by foulants. Figure 15 quantifies the coverage percentages of the samples’ surfaces (coated and uncoated) by micro- and macro-organisms associated with marine fouling.

The coated samples exhibit effective suppression of macro- and micro-colonization for the majority of micro-organisms until the 17th week of immersion in the corresponding marine environment. The antifouling coating demonstrates efficacy as a protective payer against slime mold, since its presence remains inconspicuous and limited, even after 26 weeks of immersion. In all instances, the coverage does not surpass 30% of the surface following the 50th week of residency. The uncoated samples exhibit greater coverage percentages compared to the coated samples. The deposition rate of foulants is swift and entails both micro- and macro-colonization, especially from the 17th week of immersion in seawater.

The efficiency of the antifouling coating was quantitatively assessed using the metrics of the percentage of the coating system free from biofouling organisms (fouling rate, FR) and the percentage of the coating surface area impacted by physical damage (physical damage rate, PDR). The findings from all three locations are displayed in Table 7.

The variation in the antifouling efficacy of the coating system indicates that the lack of macrofouling results in exceptionally high FR rates. The simplicity of detaching the accumulated microfouling post-rinsing of the samples indicates the anti-adhesive (or anti-stick) characteristics of the antifouling coating, as evidenced by the recorded maximum value (100%) of the PDR parameter. An effective antifouling system is defined by an OP value exceeding 80%, indicating that a biocide-free antifouling coating is adequate for the protection of naval steels.

## 4. Discussion

The experimental findings of this study indicate that the antifouling coating serves as a protective anticorrosive and antifouling layer on naval steel samples. The antifouling coating consists of two distinct nanocomposites: PAni/TiO_2_ and MWCNTs/Fe_3_O_4_. Each component contributes to the antifouling coating, unique characteristics when immersion in either artificial or natural seawater.

During the preliminary stages of immersion (immersion duration < 8 weeks), the antifouling coating preserves its thickness (Figure 12). The results from the TEM examination (Figure 9) indicate that the titania-decorated polyaniline nanorods are predominantly oriented vertically relative to the coating surface, whereas the magnetite-modified MWCNTs exhibit a horizontal alignment. The notable water contact angle values of the antifouling coating during the initial immersion stages (Figure 12) corroborates its hydrophobic properties, which may be ascribed to either PAni or MWCNTs [59,60]. Nevertheless, the modification of MWCNTs with magnetite and the comprehensive coating of their surfaces with magnetic nanoparticles (Figure 8) suggest that the hydrophobicity is attributed to the presence of the PAni, in the absence of any externally applied magnetic field [61].

The polyaniline is in its conductive state, as verified by four-probe conductivity measurements on PAni/TiO_2_, Conductive Atomic Force Microscopy (C-AFM) measurements in the antifouling coating (Figure 10) and FTIR spectrum of the PAni/TiO_2_ nanocomposite (Figure 5). The PAni’s electronic conductivity is sustained for an extended period, as the presence of TiO_2_ on the exterior of the PAni nanotubes and its doping with HCl inhibit the dissolution of anionic impurities of the conducting polymer in seawater [62].

Primary colonization foulants are defined by an accumulated negative electrostatic charge, attributed to the presence of macromolecules, phosphoryl, and carboxylic substituents in their external layer [63]. When attempting to bond with the antifouling coating, the electrostatic attraction between the matrix and foulants diminishes, ideally preventing the initiation of biofouling (Figure 15). Thus, the conductive properties of polyaniline impart antifouling features to the matrix and, consequently, to the hydrophobic coating (Figure 12).

As the duration of exposure to seawater extends, the resin dissolves, exposing portions of the PAni/TiO_2_ nanorods. The application of TiO_2_ on the polymer’s surface not only improves the conductivity characteristics of PAni, as previously noted, but also functions as a semiconducting n-type oxide that serves as an active catalyst for photocatalytic activities, leading to the in situ generation of H_2_O_2_ [64]. The non-toxic photocatalytic TiO_2_ enables the production of eco-friendly antifouling compounds that swiftly breakdown into H_2_O and O_2_ [65,66]. Consequently, during the preliminary phases of corrosion in laboratory settings, the corrosion rates in coated samples are notably minimal (Table 6). The photocatalysis mechanism is expected to be weaker in the submerged samples within the marine environments of Rafina, Kalamata, and Andros, due to the absence of diffuse light at the depths where the coated samples were situated.

As parts of the titania, noted for its hydrophilic properties, are revealed, the water contact angle values begin to diminish but remains above 90 degrees. Consequently, the surface of the antifouling coating remains hydrophobic and consistently demonstrates poor wettability. Consequently, the resin matrix’s water absorption is reduced, and its solubilization transpires in a controlled manner, thereby prolonging the durability of the antifouling coating even in static settings (Table 3, Figure 14 and Figure 15).

Following the 17th week in seawater, the coating’s thickness has diminished (Figure 12). The resin’s dissolution exposes sections of the PAni/TiO_2_ nanorods as well as the MWCNTs/Fe_3_O_4_ nanocomposite, hence decreasing the water contact angle values (Figure 12) due to the existence of the hydrophilic magnetite nanoparticles. A decrease in the contact angle enhances wettability and subsequently accelerates the dissolving rate of the resin. Following the removal of the resin, any precipitated corrosion products adhering to its surface are also eliminated, revealing a pristine layer of “virgin” coating (Figure 11). This newly exposed layer is characterized by a reduced surface corrosion rate, even after extended exposure to ASW (Figure 13, Figure 14 and Figure 15).

The incorporation of MWCNTs/Fe_3_O_4_ in their horizontal crosslinking (Figure 9) enhances the mechanical strength of the antifouling coating, ensuring compatibility with the steel’s substrate (Figure 11) and providing robust barrier properties to the antifouling coating (Figure 15), while minimizing water permeability to the steel surface (low CR values in Table 3).

Table 8 summarizes the contribution of each individual nanocomposite to the final antifouling coating.

The significant reduction in corrosion rates demonstrates the effectiveness of the coating in preventing corrosion relative to the untreated specimen (Figure 13). The specified coating system is notable for its absence of a priming layer, which is often present in commercial coatings to facilitate adhesion and bolster the corrosion resistance between the steel surface and the antifouling paint [11,67,68].

The coating’s performance during static immersion in seawater exhibits a consistent rate of 99%, as per a quantitative model established in international standards (Table 7). This efficiency, while elevated, was noted in marine habitats characterized by mild to intermediate biofouling pollution. Nonetheless, it is crucial to note that while the degree and nature of pollution in the uncoated samples were consistent at each time interval throughout the 50-week trial, the coated samples shown remarkable resistance to macrofouling overlap and exhibited distinct behavior in addressing microfouling (Figure 15).

Thus, the results under submerged site conditions reveal that the antifouling efficacy of the coating exhibits remarkable persistence of the coated naval steel over an extended period. The multifunctional coating effectively addresses the needs for antifouling protection while simultaneously providing corrosion protection for the metal substrate.

Table 9 presents a comparison of the coating’s functioning with commercially available antifouling coatings in the Greek market, which were either subjected to immersion in the marine environment of Rafina or laboratory-corroded in a 3.5% NaCl solution [17].

The antifouling coatings demonstrate superior performance compared to their commercial equivalents, both in laboratory and natural immersion settings. It is important to acknowledge that the size of the coated samples utilizing commercial antifouling coatings were greater than those in the current investigation, and the immersion process was conducted at different intervals.

The antifouling coating is environmentally safe and exhibits excellent antifouling efficacy, durability, and mechanical resistance, in accordance with the criteria specified in Table 2. Despite its ease of application, the formulation process is intricate and necessitates specialized skills, resulting in somewhat expensive production costs.

Nonetheless, it is essential to evaluate the long-term efficacy of the antifouling coating in hostile (aggressive) maritime settings, where suboptimal broad-spectrum performance may manifest. Comprehensive field testing across diverse water, temperature, and pH conditions is essential to assess the coating’s degradation over time. Furthermore, the intricacy of producing the coating system must be alleviated to ensure that the production of the refining system remains efficient and economical. It is also essential to verify compliance with stringent environmental safety standards to mitigate any long-term environmental consequences.

## 5. Conclusions

This study illustrates the efficacy of an antifouling coating on naval steel specimens, comprising two separate nanocomposites: PAni/TiO_2_ and MWCNTs/Fe_3_O_4_. The findings indicate that the antifouling coating functions as a protective anticorrosive barrier and antifouling layer on the surface of the naval steel samples. As the coating thickness decreases during static immersion, precipitated corrosion products are removed, revealing a new layer of “virgin” coating. This new layer demonstrates a reduced surface corrosion rate despite extended exposure to artificial seawater. The absence of porosity and retention of adhesion enhance the corrosion resistance of the coating and confirm the barrier properties of the antifouling paint.

Following one week of exposure to ASW, the corrosion rate of 21.401 mm/y in the uncoated specimen is diminished to zero in the coated specimen, signifying a 100% reduction. The significant reduction in the corrosion rate is maintained after 2 weeks of exposure, reaching a value of 99.986%. The coating demonstrates a continuous performance rate of 99% during static immersion in seawater. The coated samples exhibited notable resistance to macrofouling overlap and diverse responses to microfouling.

The conductive characteristics of polyaniline impart antifouling attributes to the hydrophobic coating. As the exposure time to seawater extends, the resin dissolves, revealing portions of the PAni/TiO_2_ nanotubes. The application of TiO_2_ on the polymer surface enhances the conductivity properties of PAni and serves as an active catalyst for photocatalytic processes. The functionalized MWCNTs with Fe_2_O_3_ provide anti-adhesion capabilities to the coating and enhance its cohesion with the steel substrate.

The antifouling coating is ecologically safe and demonstrates superior antifouling effectiveness, longevity, and mechanical resilience over a prolonged duration of immersion. The antifouling coating efficiently fulfills the requirements for antifouling protection while concurrently offering corrosion resistance for the metal substrate.

Nonetheless, comprehensive field testing under different sea sides, temperature, and pH conditions is necessary to evaluate the coating’s deterioration over time. The complexity of manufacturing the coating system must be simplified to provide efficient and cost-effective production, but adherence to rigorous environmental safety requirements is crucial to prevent long-term ecological impacts.

## 6. Patents

There is one patent resulting from the work reported in this manuscript: WO2024224120A1, Electrically anisotropic antifouling coatings, BFP Advanced Technologies, 2024 [37].

## Figures and Tables

**Table 1:** Antifouling strategies on ship hulls and their advantages and disadvantages.

Period	Technology	Advantages	Disadvantages
<1800 A.D.	Pitch, organic residues (oil), copper foil	Natural protection, easy application	Limited durability, environmentally unstable
1800–1950	Copper and lead-based marine paints	Effective, commercially available	Toxic to aquatic organisms
1960–2000	Paints with tributyltin (TBT)	Very high effectiveness	Very toxic Worldwide ban in 2008
2000–today	Self-polishing copolymers (SPCs)	Controlled release of biocides, stable performance	Still contain copper or other mild biocides
2010–today	Silicone-based/fluoropolymer-based hydrophobic paints	Biocide free, very smooth surface, environmentally friendly	More precise, require high surface preparation
2015–today	Biomimetic surfaces (e.g., Sharklet^TM^, Aurora, CO, USA)	Chemical-free antifouling action	In development—cost, durability
2020–today	Robotic cleaners (π.χ. HullWiper (Dubai, United Arab Emirates), ECOsubsea (Torangsvåg, Norway))	No chemicals, repeated use	Requires frequent application—limitations in ports
2020–today	UV illumination/electrochemical methods	Fully non-chemical solutions	Experimental stage

**Table 3:** Designation of naval steel samples in static immersion tests.

	Sample Notation for Laboratory Tests	Sample Notation for In Situ Tests	Immersion Time (Weeks)
Uncoated samples	L00	S00	0
	L01	S01	1
	L02	S02	2
	L03	S03	8
	L04	S04	17
	L05	S05	26
	L06	S06	50
Coated samples	L0	S0	0
	L1	S1	1
	L2	S2	2
	L3	S3	8
	L4	S4	17
	L5	S5	26
	L6	S6	50

**Table 4:** Identification of FTIR peaks of PAni/TiO_2_ nanocomposite.

Peak’s Number	Peak’s Wavenumber	Identification of Peak	Ref.
1	3407 cm^−1^	N–H stretching bands of PAni	[44]
2	3230 cm^−1^	C–H stretching bands PAni	[44]
3	1567 cm^−1^	C=C stretching vibrations of quinoid ring of PAni	[45]
4	1487 cm^−1^	C=C stretching vibrations of benzenoid ring of PAni	[45]
5	1298 cm^−1^	C–N stretching modes of the benzenoid ring of PAni	[46]
6	1246 cm^−1^	C–N stretching modes of the benzenoid ring of PAni	[46]
7	816 cm^−1^	C–H out of plane bending vibrations of PAni	[47]
8	1141 cm^−1^	C–H in plane bending vibrations of PAni	[48]
9	647 cm^−1^	Ti–O–Ti stretching mode of anatase (TiO_2_)	[49]

**Table 5:** Identification of FTIR peaks of MWCNTs/Fe_3_O_4_ nanocomposite.

Peak’s Number	Peak’s Wavenumber	Identification of Peak	Ref.
1	1620 cm^−1^	aromatic C=C stretching vibrations	[52]
2	1400 cm^−1^	carboxylic functionalized group	[53]
3	597 cm^−1^	Fe–O–Fe stretching vibrations	[54]

**Table 6:** The parameters of weight change and corrosion rate for coated and uncoated steel specimens after various immersion durations in ASW.

Sample Notation	Immersion Time t (Weeks)	Initial Weight wi (g)	Final Weight wf (g)	Weight Change W = wi − wf (g)	Weight Change (w/wi) × 100 (%)	Corrosion Rate CR (mm/Year)
L00	0	126.425 ± 0.004	126.425 ± 0.006	0.000 ± 0.007	0.000	-
L01	1	126.267 ± 0.007	121.158 ± 0.004	5.109 ± 0.008	4.046 ± 0.002	21.401 ± 0.263
L02	2	126.419 ± 0.009	119.259 ± 0.007	7.160 ± 0.011	5.664 ± 0.002	14.996 ± 0.186
L03	8	126.697 ± 0.008	108.369 ± 0.005	18.328 ± 0.009	14.466 ± 0.001	9.597 ± 0.038
L04	17	126.694 ± 0.007	101.236 ± 0.009	25.458 ± 0.011	20.094 ± 0.001	6.273 ± 0.022
L05	26	126.412 ± 0.009	99.269 ± 0.007	27.143 ± 0.011	21.472 ± 0.000	4.373 ± 0.014
L06	50	126.159 ± 0.008	98.369 ± 0.003	27.790 ± 0.009	22.028 ± 0.000	2.328 ± 0.006
L0	0	132.759 ± 0.008	132.759 ± 0.008	0.000	0.000	-
L1	1	132.459 ± 0.004	132.459 ± 0.009	0.000	0.000	0.000
L2	2	132.691 ± 0.000	132.690 ± 0.000	0.001 ± 0.000	0.001 ± 0.000	0.002 ± 0.000
L3	8	132.698 ± 0.000	132.696 ± 0.000	0.002 ± 0.000	0.002 ± 0.000	0.001 ± 0.000
L4	17	132.459 ± 0.000	132.449 ± 0.000	0.010 ± 0.000	0.008 ± 0.000	0.002 ± 0.000
L5	26	132.784 ± 0.002	131.525 ± 0.003	1.259 ± 0.004	0.948 ± 0.003	0.203 ± 0.007
L6	50	132.745 ± 0.001	130.476 ± 0.004	2.269 ± 0.004	1.709 ± 0.002	0.190 ± 0.005

**Table 7:** Summary of the fouling rate and physical damage rate of antifouling coating.

Locations	Parameters	S0	S1	S2	S3	S4	S5	S6
Rafina	FR (%)	100	100	99	99	99	99	99
	PDR (%)	100	100	100	100	100	100	100
	OP (%)	100	100	99	99	99	99	99
Kalamata	FR (%)	100	100	100	99	99	99	99
	PDR (%)	100	100	100	100	100	100	100
	OP (%)	100	100	99	99	99	99	99
Andros	FR (%)	100	100	99	99	99	99	99
	PDR (%)	100	100	100	100	100	100	100
	OP (%)	100	100	99	99	99	99	99

**Table 8:** Impact of the nanocomposites to the antifouling performance.

Material/System	Key Properties	Antifouling Mechanism	Advantages
PAni	Conductive	Disrupts biofoulant cell walls through redox cycling	Corrosion protection
TiO_2_ (anatase)	Photocatalytic	Generates reactive oxygen species (OH^•−^, O_2_^•−^)	
MWCNTs	Conductive	Anti-adhesion	Mechanical strength, electrical conductivity
Fe_3_O_4_	Magnetic	Antibacterial	
PAni/TiO_2_	Conductive + photocatalytic	reactive oxygen species generation	enhanced corrosion resistance
MWCNTs–Fe_3_O_4_	Conductive + magnetic	Anti-adhesion	enhanced corrosion resistance
PAni–TiO_2_–MWCNTs–Fe_3_O_4_	All-in-one system	Physical barrier	Broad-spectrum antifouling, corrosion resistance, mechanical strength

**Table 9:** Comparative analysis of the behavior of the antifouling coating vs. commercially available alternatives. Comparisons were conducted under analogous static laboratory and actual immersion settings.

Antifouling Coating	Location	Conditions	Performance
Cooper biocide release	Rafina	In situ	9-month coverage = 48%
	Laboratory	3.5% NaCl	maximum CR = 0.340 mm/y
Self-polishing copolymer	Rafina	In situ	9-month coverage = 92%
	Laboratory	3.5% NaCl	maximum CR = 1.600 mm/y
Gradual polishing paint	Rafina	In situ	9-month coverage = 92%
	Laboratory	3.5% NaCl	maximum CR = 2.600 mm/y
This work	Rafina	In situ	9-month coverage = 30%
	Laboratory	ASW	maximum CR = 0.203 mm/y

## Data Availability

The original contributions presented in this study are included in the article. Further inquiries can be directed to the corresponding author.

## Abbreviations

The following abbreviations are used in this manuscript:

AF	antifouling
TBT	tributyltin
CNTs	carbon nanotubes
PAni	polyaniline
NPs	nanoparticles
ASW	artificial seawater
SEM	scanning electron microscopy
FR	Foul Resistance
PDR	Physical Data Rating
OP	Overall Performance
CR	corrosion rate
